# Looking under the hood of a hybrid two-way texting intervention to improve early retention on antiretroviral therapy in Malawi: an implementation fidelity evaluation

**DOI:** 10.21203/rs.3.rs-4965561/v1

**Published:** 2024-12-19

**Authors:** Robin Klabbers, Caryl Feldacker, Jacqueline Huwa, Christine Kiruthu-Kamamia, Agness Thawani, Hannock Tweya

**Affiliations:** University of Washington; University of Washington; Lighthouse Trust; Lighthouse Trust; Lighthouse Trust; International Training and Education Center for Health

**Keywords:** mobile health, digital health innovation, interactive messaging, short message service, implementation science, early retention in antiretroviral therapy, routine settings, Malawi, implementation fidelity, framework

## Abstract

**Background:**

While key to interpreting findings and assessing generalizability, implementation fidelity is underreported in mobile health (mHealth) literature. We evaluated implementation fidelity of an opt-in, hybrid, two-way texting (2wT) intervention previously demonstrated to improve 12-month retention on antiretroviral therapy (ART) among people living with HIV (PLHIV) in a quasi-experimental study in Lilongwe, Malawi.

**Methods:**

Short message service (SMS) data and ART refill visit records were used to evaluate adherence to 2wT content, frequency and duration through the lens of the Conceptual Framework for Implementation Fidelity. Message delivery and 2wT participant interactions were considered across four core 2wT components: 1) weekly motivational SMS messages; 2) proactive SMS appointment reminders; 3) SMS reminders after missed appointments; and 4) interactive messaging with 2wT staff about transfers and appointment rescheduling. In mixed-effects logistic regression, associations were assessed between 2wT messages and on-time appointment attendance and timely return to care after a missed appointment, respectively, presenting odds ratios (ORs) and 95% confidence intervals (CIs).

**Results:**

The 468 2wT participants had a median of 52 study weeks (interquartile range [IQR] 34–52) with 6 ART appointments (IQR 4–7) of which 2 (IQR 1–3) were missed. On average, participants received a motivation message for 75% (IQR 56% − 83%) of enrolled weeks, a reminder before 83% (IQR 67% − 100%) of appointments, and after 67% (IQR 0% − 100%) of missed appointments. Participants reported 9 transfers and rescheduled 46 appointments through 2wT prompts; 196 appointments were changed via unprompted interaction. Participants with 10% higher expected motivation message delivery were more likely to attend clinic on time (OR: 1.13; 95%CI: 1.06–1.20, p < 0.001) and return to care within 14 days of a missed appointment (OR: 1.12; 95%CI: 1.03–1.22, p = 0.01). Receiving an appointment reminder increased on-time attendance (OR: 1.83; 95%CI: 1.44–2.33, p < 0.001).

**Conclusion:**

Greater 2wT implementation fidelity was associated with improved care outcomes. Although implementation fidelity monitoring of mHealth interventions is complex, it should be integrated into study design.

## BACKGROUND

Fidelity refers to the degree to which an intervention is implemented as intended.^[1]^ It provides a plausible link between intervention efficacy and intended outcomes and is key to distinguishing intervention design failure from implementation failure.^[2]^ Implementation fidelity is highly correlated with intervention success: programs implemented with high fidelity have been demonstrated to perform better than poorly implemented programs.^[3–11]^ Reporting on fidelity is necessary for others to evaluate whether findings can reliably be attributed to the described intervention, to be able to replicate results in a new setting, and to establish the minimum dose of an intervention that is required to produce a desired change. Despite its importance, and the existence of several guidelines for digital health research that prescribe transparent and rigorous fidelity reporting,^[12–14]^ implementation fidelity is largely underreported in the electronic health (eHealth) and mobile health (mHealth) literature.^[15]^ A systematic review of mHealth interventions for physical activity using the RE-AIM framework highlighted that less than a quarter of the 15 included trials reported details on intervention implementation, and only two trials commented on implementation fidelity, specifically.^[16]^ A second systematic review synthesizing the implementation outcomes of mHealth interventions for the prevention of HIV and sexually transmitted infections among young people in low- and middle-income countries (LMICs) found similar gaps: fidelity was examined explicitly for only one of the six included interventions.^[17]^ Across both systematic reviews, authors emphasized that reporting on the degree to which an intervention is delivered as intended is needed to replicate findings and assess generalizability. More details on mHealth implementation fidelity would facilitate more effective intervention planning, evaluation, and optimization.

Findings from mHealth fidelity studies provide insight into why implemented interventions did or did not work and reveal ways into which the intervention or its implementation could be adapted to be more effective in the study setting. A process evaluation of a randomized-controlled trial in Kampala, Uganda investigating the ability of one-time short message service (SMS) text messaging to relay the results of tuberculosis screening and promote the uptake of follow-up services found low implementation fidelity: SMS messages were sent out to only 58% of eligible participants as a result of programming errors, server-related issues, and missing participant information.^[18]^ Of those participants to whom messages were sent out, only 67% received and only 52% confirmed reading the SMS message, with network outages, phone malfunctioning and phone sharing practices offered as potential explanations.^[18]^ The detailed cascade analysis uncovered unobserved barriers that together resulted in a cumulative likelihood of receiving, reading, and retaining SMS messages of only 19%. Another process evaluation of an mHealth antiretroviral therapy (ART) adherence intervention consisting of individualized voice calls and SMS text messages noted similar challenges which resulted in only 22% and 72% of participants receiving the voice or SMS intervention, respectively.^[19]^ Combined, these process evaluations strongly suggest that mHealth interventions require rigorous evaluation of implementation fidelity.

Several frameworks evaluate implementation fidelity.^[2, 6, 20–22]^ Since 2007, the Conceptual Framework for Implementation Fidelity (CFIF), developed by Carroll et al.,^[2]^ has widely been used to assess implementation fidelity including for interventions related to fall prevention,^[23]^ medication adherence,^[24]^ occupational health,^[25]^ health care decision-making,^[26]^ assisted partner notification for HIV,^[27]^ tuberculosis screening,^[28]^ and maternal health.^[29]^ In the CFIF, assessing fidelity is conceptualized as measuring *adherence*, a concept composed of the elements *content*, *coverage*, *frequency*, and *duration*. *Adherence to content* can be interpreted as whether the ‘active ingredients’ or core intervention components have been delivered to participants. *Adherence to coverage* refers to whether all individuals eligible to receive the intervention participated and received its benefits. *Adherence to frequency and duration*, together, capture whether the ‘dose’ at which the intervention was delivered matched intervention protocols, i.e., did participants receive the intervention as often and/or for as long as intended? According to the CFIF, the level of adherence to content, coverage, frequency, and duration is influenced by several factors, termed ‘moderators’ in the framework, which include *intervention complexity*, *facilitation strategies*, *quality of delivery*, and *participant* (and deliverer) *responsiveness*.

We used the CFIF to evaluate the implementation fidelity of an opt-in, hybrid, two-way texting (2wT) intervention to improve 12-month retention on ART among new ART initiates that was implemented in a quasi-experimental study at Martin Preuss Centre (MPC) in Lilongwe, Malawi. Prior effectiveness analysis demonstrated that clients receiving 2wT had a 62% lower hazard of dropping out of ART care than clients receiving standard of care at any point during the twelve month follow up period.^[30]^ In the current analysis, we ‘look under the hood’ of this quasi-experimental 2wT study and explore whether the 2wT intervention was delivered as intended with the objective to: 1) deepen our understanding of intervention effectiveness; 2) identify areas for implementation improvement during future 2wT expansion; and 3) generate hypotheses about ‘essential’ intervention components. Reporting the degree to which the 2wT intervention was delivered as intended is best practice and will promote the generalizability of our findings.

## METHODS

### Setting

The quasi-experimental 2wT study was conducted at MPC, an urban flagship Lighthouse Trust (LT) clinic offering comprehensive HIV services including ART to over 38,000 people living with HIV (PLHIV) in Lilongwe Malawi. At LT clinics, including MPC, individuals newly diagnosed with HIV are initiated on ART following the test and treat strategy. A point-of-care electronic medical record system (EMRS) is used by healthcare providers to manage clients’ data and manage their care. At ART registration, phone numbers are captured in the EMRS and locator forms are filled out for new ART initiates who consent to be traced, if needed. During the first six months on ART, ART clinic visits are scheduled monthly after which, if the client is stable and adherent to ART, visits are scheduled at three- or six-month intervals.

### Quasi-experimental study

The 2wT intervention was designed, implemented and evaluated in a quasi-experimental study conducted between August 2021 and June 2023 (see TREND Statement Checklist in Additional file 1).^[30]^ Adult clients ages 18 and older who initiated ART at MPC < 6 months prior, with basic literacy (ability to read a provided example text message and enter the correct response to an example appointment reminder) and in possession of a basic mobile phone, were recruited and those opting-in to participation were consented. After confirming receipt of a 2wT enrolment text, 2wT clients were enrolled in the study for twelve months unless they requested to stop receiving messages, transferred care to a different health center, were lost to follow-up, stopped ART, or died. A comparison cohort was identified through random selection from a database of ART clients with phone numbers who had received standard of care (SoC) at MPC one year prior to the intervention. SoC clients were matched 1:1 to 2wT clients on age (in bands of 5 years), sex, and World Health Organization (WHO) stage at ART initiation. The 468 2wT and 468 SoC participants were followed during their first year on ART and retention on ART twelve months post-ART initiation was compared between the two groups.

### 2wT intervention

**Figure 1.**
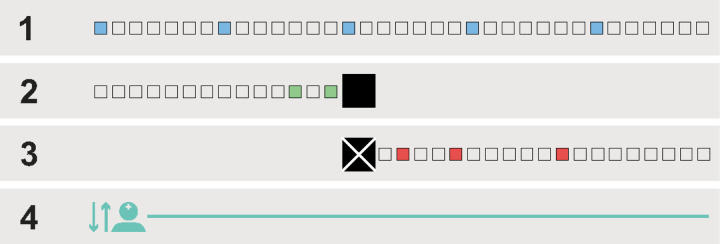
Core intervention components 1. Weekly automated motivation SMS messages; 2. Proactive, automated SMS appointment reminders; 3. Automated SMS missed appointment reminders; 4. Opportunity to interact with a health worker through SMS or phone call to reschedule appointments and report transfers.

2wT technology was developed using the open-source Community Health Toolkit (CHT).^[31]^ The co-design and pilot-testing of the 2wT intervention to promote early retention on ART have previously been described.^[32, 33]^ The 2wT intervention implemented in the quasi-experimental 2wT study at MPC consisted of four main, or ‘core’, components (Fig. 1): 1) automated weekly motivation SMS text messages with non-HIV-related content (e.g., “You are taking care of your health” and “Drink boiled or chlorinated water, 2 to 4 liters a day”); 2) Proactive SMS appointment reminders 3 days and 1 day before an appointment, which were muted upon confirmation of intent to attend the appointment; 3) SMS missed appointment reminders 2, 5, and 11 days after missing an appointment, which were deactivated upon confirmation of intent to reschedule; and 4) the ability to interact with a health worker through text to reschedule appointments, report transfers or to request a phone call. The 2wT intervention required only a basic phone with texting and receiving capabilities. Both sending and receiving SMS within the 2wT system was free for participating clients.

### Data sources

SMS data including message delivery status (success, failure, sent, rejected, or expired) were obtained from the 2wT database and Africa’s Talking, the SMS aggregator. Information on visit attendance was extracted from MPC’s EMRS. Additionally, implementation notes kept by study staff throughout the study were reviewed to provide context.

### Definitions

Message delivery status was categorized as ‘success’ if the message was delivered to the participant’s mobile phone. All other statuses indicated that message delivery was non-successful: ‘failure’ indicated the message was not delivered; ‘sent’ indicated the message was sent but no final status was available; ‘rejected’ indicated the message was not accepted for delivery by the mobile operator; and, ‘expired’ indicated the network was unable to send the message because the phone was either switched off or out of signal for 48 hours.

### Analysis

#### Assessment of implementation fidelity

Guided by the CFIF, we evaluated implementation fidelity of the 2wT intervention by assessing adherence to intervention content (to what extent were 2wT core components received?), and frequency and duration (were core components received consistently throughout follow-up?). The fourth element of adherence - coverage (reach) - was previously reported together with effectiveness outcomes.^[30]^ In the quasi-experimental study evaluating the 2wT intervention, 44% of the clients screened were reached by the intervention, having met 2wT eligibility criteria including basic literacy and phone access.^[30]^

For the first three intervention core components, we calculated message delivery success per participant by dividing the number of delivered messages by the number of expected messages. The median across all participants was used to determine overall message delivery success per message type (Appendix 1). In addition, totals of expected, transmitted, and delivered messages were reported per message type (Box 1). Differences in motivation message delivery success and proactive appointment reminder delivery success were explored by age (< median age of 33 years vs ≥ median age of 33 years) and sex, respectively, using Mann-Whitney U tests. For core component 1, we plotted the expected total number of motivation messages per study week categorizing these messages by sent and delivery status. Heterogeneity in motivation message delivery patterns between participants was explored visually in a heat map displaying motivation message transmission and delivery status per week from study enrolment until a study outcome was reached for each participant (Appendix 2). We used survival analysis to assess the continuity of motivation message delivery, reporting the median time until the first motivation message delivery gap of ≥ 3 consecutive weeks (failure event). This motivation message delivery gap threshold was selected as a representation of an interruption in communication that could plausibly influence care engagement. In this analysis, reaching a study outcome (study withdrawal, death, transfer out, stopping ART, and reaching 12 months on ART) was considered a censoring event. For core component 2, we plotted proactive appointment reminder transmission and delivery success per appointment event (e.g., first appointment, second appointment, third appointment) and visually assessed trends in the plotted data. For core component 3, we plotted missed appointment reminder transmission and delivery success by missed appointment event (e.g., first missed appointment, second missed appointment, third missed appointment) and visually assessed trends in the plotted data. The distribution of the number of days by which appointments were missed was explored. For core component 4, we evaluated 2wT interactions related to care transfer, visit rescheduling, and confirming appointment attendance. We tallied the number of transfer reports sent by participants in response to automated 2wT prompts and calculated the percentage of cases for which, upon verification, a true transfer had occurred. Of the participants with ‘transfer out’ as 12-month study outcome, we assessed the percentage that had reported this transfer through 2wT. We tallied the number of rescheduling requests sent in response to 2wT prompts and spontaneously through 2wT, respectively, and calculated the percentage of these cases for which rescheduling took place. Per appointment and missed appointment event (e.g., first, second, third, etc), participant response was plotted.

To evaluate the merit of multiple reminder messages, we assessed the degree to which participants responded to subsequent reminder messages for an appointment if they left the first reminder message unanswered. For participants who withdrew from the study, we assessed the median length of time until they requested messages to be muted. We tallied the number of participants who communicated a phone number change during the study period and reported the reason for the change.

#### Effect of core component implementation on clinic attendance

Univariable mixed-effects logistic regression models accounting for clustering at the participant level through random intercepts were used to assess the association between: a) the percentage of enrolled weeks for which motivation messages were delivered (core component 1) and on-time clinic attendance (attendance on the scheduled appointment date); b) the percentage of enrolled weeks for which motivation messages were delivered (core component 1) and return to care within 14 days of a missed appointment; c) proactive appointment reminder receipt (core component 2) and on-time clinic attendance; d) missed appointment reminder receipt (core component 3) and return to care within 14 days of a missed appointment; e) response (any answer) to a delivered appointment reminder (core component 4) and on-time clinic attendance; and, f) response (any answer) to a delivered missed appointment reminder (core component 4) and return to care within 14 days of a missed appointment. Additionally, in multivariable mixed-effects logistic regression, models c – f were adjusted for the percentage of enrolled weeks for which motivation messages were delivered (core component 1). For models looking at the effect of proactive appointment reminders and missed appointment reminders, appointments for which no reminders were expected were excluded from the analysis (i.e., appointments attended > 3 days early or missed by < 2 days). Odds ratios (ORs) and 95% confidence intervals (CIs) were reported for all models. Analyses and visualizations were performed using R (version 4.3.1) and RStudio.^[34]^

## RESULTS

A total of 468 participants opted-in and were exposed to the 2wT intervention. Participants were followed during their first year on ART and spent a median of 52 weeks (interquartile range [IQR] 34–52) in the study. Table 1 summarizes implementation fidelity by 2wT core component.

### Core component 1: Participant receives weekly motivation messages

As designed, participants spent a median of 52 weeks (IQR 34–52) in the study, in which 52 motivation messages were expected. In practice, the 2wT platform sent out motivation messages for 44 (IQR 30–47) enrolled weeks and messages were successfully delivered for 31 (IQR 16–42) enrolled weeks, corresponding to receiving a motivation message for 75% (IQR 56% − 83%) of enrolled weeks (Appendix 1). There was substantial heterogeneity in message delivery patterns across participants (Appendix 2). Message delivery success was higher for older (77%; IQR 62% − 85%) compared to younger participants (71%; IQR 50% − 83%) (p = 0.01) and for female (77%; IQR 60% − 85%) compared to male participants (73%; IQR 50% − 83%) (p = 0.01). Over 19,787 combined participant weeks, a motivation message was sent for 17,434 participant weeks (88% of enrolled weeks), and successfully delivered for 13,423 participant weeks (68% of enrolled weeks). Overall, message delivery was unsuccessful for 23% of transmitted motivation messages. Of the 4,012 undelivered messages, 3,200 (80%) expired, 588 (15%) had ‘sent’ status, 180 (4%) were rejected, and 44 (1%) failed.

**Figure 2.**
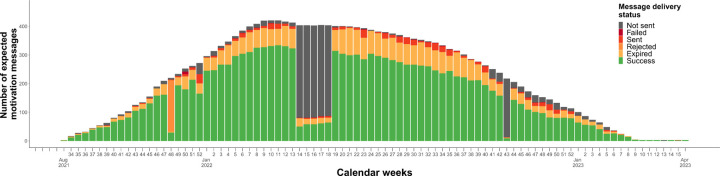
Distribution of the number of motivation messages sent by the 2wT platform over time by message delivery status

Evaluating the distribution of motivation message delivery status per week over time (Fig. 2), weeks that were clear anomalies can be identified in which a substantially lower number of messages were sent out by the platform than expected (weeks 14–18 and week 43 of 2022) or in which a substantially higher number of messages expired (week 48 of 2021).

**Figure 3.**
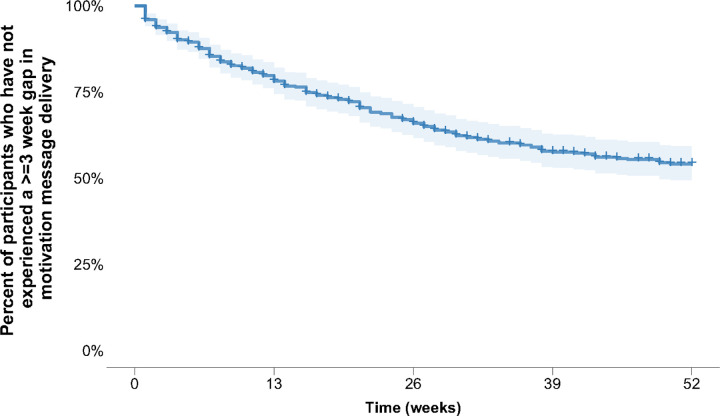
Kaplan-Meier survival plot showing time until the first occurrence of a gap of ≥ 3 weeks in motivation message delivery

In survival analysis, we found that sixty percent of participants consistently received messages and did not experience a gap of 3 consecutive weeks without motivation messages while enrolled in the study (Fig. 3). For the 190 (41%) participants who experienced undelivered motivation messages for three or more consecutive weeks, the median time to event was 13 weeks (IQR 5–25 weeks)

### Core component 2: Participant is proactively reminded of a scheduled appointment

**Figure 4.**
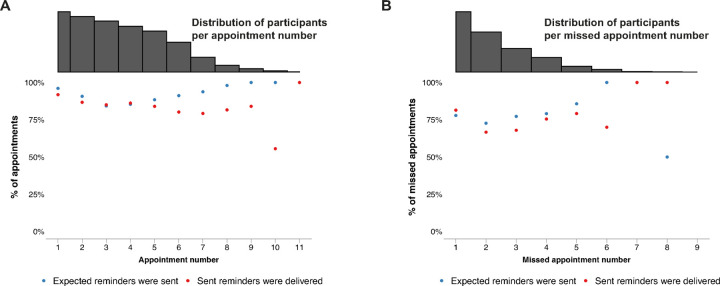
Delivery success of proactive appointment reminders and missed appointment reminders by appointment number* *Appointment interval heterogeneity explains the variation in the number of appointments participants have within the same time period.

Participants had a median of 6 (IQR 4–7) clinic appointments during the study period. They were expected to have received a proactive reminder for 5 (IQR 3–6) of these appointments (no messages were expected for appointments attended > 3 days early). Typically, the 2wT platform sent proactive reminders for 5 (IQR 3–6) clinic appointments and reminders were received by participants for 4 (IQR 2–5) appointments, an expected appointment reminder delivery success percentage of 83% (IQR 67% − 100%) (Appendix 1). Message delivery of expected proactive appointment reminders did not differ between younger and older participants or between male and female participants. Collectively, participants had 2,513 clinic appointments during the study, for 2,353 of which reminders were expected. Participants were sent proactive reminders for a total of 2,120 clinic appointments (90% of eligible appointments), and received them for 1,819 appointments (77% of eligible appointments). Of the 301 (14%) transmitted appointment reminders that were not delivered, 290 (96%) expired and 10 (3%) had ‘sent’ status. Both reminder transmission and delivery of appointment reminders were generally high (> 75%) throughout participants’ follow-up trajectory (Fig. 4A). After an initial decline, reminder transmission appeared to improve later in follow-up. Delivery of transmitted messages, on the other hand, appeared to decline over the course of follow-up. Additionally, appointment reminders were transmitted for 74 appointments and delivered for 67 (91%) appointments that did not appear in the EMRS, indicating a potential system error.

### Core component 3: Participant is reminded after a missed appointment

Participants missed a median of 2 (IQR 1–3) appointments throughout follow-up. A missed appointment reminder was expected for 1 (IQR 1–2) of these appointments (no message was expected for appointments missed by less than 2 days). Generally, the 2wT platform sent reminders for 1 (IQR 0–2) missed appointment and participants received reminders for 1 (IQR 0–1) missed appointment, an expected missed appointment reminder delivery success percentage of 67% (IQR 0% − 100%) (Appendix 1). Collectively, participants missed 868 appointments, for 606 of which, they were expected to receive missed appointment reminders (appointment missed by 2 or more days). Participants were sent reminders for 468 (77%) of these eligible missed appointments and received reminders for 349 (58%) of these eligible missed appointments. For 119 eligible missed appointments, transmitted reminders were not delivered; for 109 (92%) of them, the reminders expired and for 10 (8%), the reminders had ‘sent’ status only. Transmission of missed appointment reminders improved throughout follow-up while delivery of transmitted missed appointments decreased with missed appointment number (Fig. 4B).

For the 262 missed appointments that were missed by less than 2 days, for which no reminder message should have been sent out, missed appointment reminders were transmitted in 149 cases and delivered in 111 (74%) cases. Additionally, the 2wT platform sent out 406 missed appointment reminders and delivered 345 (85%) missed appointment reminders for appointments that had been attended by participants on or before their scheduled appointment date. Finally, missed appointment reminders were sent out for 30 appointments that were not found in the EMRS; 25 (83%) were delivered. Taken together, in 585 (56%) of the 1,053 instances in which missed appointment reminders were transmitted, they were not transmitted as intended.

### Core component 4: Participants can interact with the 2wT platform to report transfers request visit rescheduling and confirm planned visit attendance

#### Transfer

Participants who indicated that they would not return to clinic by responding with a ‘0’ to an appointment or missed appointment reminder prompt, received an automated follow-up prompt inquiring whether they had transferred. A total of 150 follow-up prompts were delivered, to which participants responded affirmatively 47 (31%) times. These participants were followed up on, and it was found that true (temporary) transfer had taken place in 9 (19%) of these cases. Of the 468 2wT intervention participants, 39 (8%) participants had ‘transfer out’ as their final study outcome at 12 months: 3 (8%) had reported this transfer via 2wT and 36 (92%) had communicated this through other means (e.g., during clinic visits, in a call with 2wT staff, or reported transfer when traced).

#### Rescheduling

Throughout the study, 326 requests were made to reschedule clinic appointments. Of these requests, 115 requests (35%) were made by 82 participants (18%) in response to 2wT rescheduling prompts, and 211 requests (65%) from 153 participants (33%) were spontaneous requests. Of the 115 prompted rescheduling requests, 46 (40%) resulted in appointment changes. The 211 spontaneous requests resulted in 196 (93%) appointment changes.

#### Visit attendance confirmation

**Figure 5.**
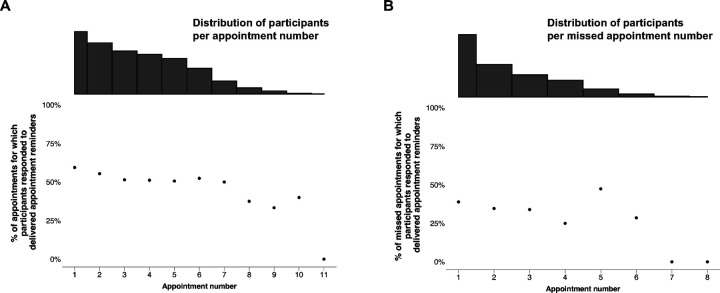
Participant response to successfully delivered (missed) appointment reminders by (missed) appointment number* *Appointment interval heterogeneity leads to variation in the number of appointments participants have within the same time period.

To explore participant responsiveness throughout follow-up, the percentage of appointments and missed appointments for which participants responded to delivered reminder messages was plotted per appointment and missed appointment number (first, second, third, etc), respectively (Figs. 5A & 5B). For both proactive appointment reminders and missed appointment reminders, participant responsiveness appeared to decrease over time.

We evaluated the response to subsequent proactive appointment reminders for participants who did not respond to the initial reminder. Among participants who did not respond to a reminder sent three days before a scheduled visit, but received a second reminder one day before the visit, 26% (237/921) responded to the second appointment reminder. A similar analysis was conducted for missed appointment reminders. Of the participants who did not respond to a successfully delivered reminder two days after their missed appointment, but received a second reminder three days later, 15% (8/54) responded to the second reminder. Among those who did not respond to the second reminder but received a third reminder eleven days after their missed appointment, 17% (8/48) responded to the last reminder.

#### Message muting and phone number changes

During the study period, 2wT participants could request to stop receiving motivation messages and/or reminders. Of the 468 intervention participants, 20 (4%) participants requested to mute messages, with 16 (75%) wanting both message types muted, and 4 (25%) requesting to mute motivation messages only. The average time to motivation message muting was 39 days (IQR 20–77 days) and to reminder muting was 48 days (IQR 15–78 days). During the study, 16 (3%) participants provided a second phone number to the study team. For 14 (88%) participants, this was an alternative/additional number and for 2 (13%) participants this concerned a phone number change.

#### Effect of core component implementation on clinic attendance

For every 10% more successful delivery of expected motivation messages, participants had 1.13 (95% CI: 1.06–1.20, p < 0.001) times higher odds of attending clinic on the scheduled date (Table 2). After controlling for receiving a proactive appointment reminder, this association remained significant (OR 1.11, 95%CI: 1.03–1.19, p = 0.004). A 10% delivery increase was also associated with higher odds of returning to care within 14 days of a missed appointment (OR 1.12, 95%CI: 1.03–1.22, p = 0.01), though this was no longer significant after controlling for receiving a missed appointment reminder. Having a successfully delivered appointment reminder (compared to not) was associated with a 1.83 times higher odds (95%CI 1.44–2.33, p < .001) of attending that appointment on the scheduled date. This association remained significant after controlling for the percentage of expected motivation messages that were delivered (OR 1.65, 95%CI: 1.29–2.12, p < 0.001). Among participants with true missed appointments, having a successfully delivered missed appointment reminder (compared to not) was associated with a 1.33 times higher odds (95%CI: 0.91–1.95, p = 0.14) of returning to care within 14 days of a missed appointment, although this was not significant. Those who responded to a delivered proactive appointment reminder had a 1.52 time higher odds (95%CI: 1.19–1.95, p < 0.001) of attending the appointment on the scheduled date compared to those who did not respond. Those who responded to a delivered missed appointment reminder had a 1.48 times higher (95%CI 0.86–2.55, p = 0.15) odds of returning to care within 14 days compared to those who did not respond.

## DISCUSSION

Our assessment of the implementation fidelity of a 2wT intervention to improve ART retention among new initiates in a routine ART setting in Malawi found good adherence to intervention content and dose. On average, participants received weekly motivation messages for 75% of weeks that they were enrolled, a text message reminder before 83% of scheduled clinic appointments, and a text message reminder after 67% of missed appointments. Interactive features of 2wT were successfully used by a minority of participants to report transfers and reschedule appointments. 2wT participants receiving reminder messages as expected exhibited 83% higher odds of attending scheduled clinic appointments compared to those who did not receive a reminder message. Actively engaging with 2wT proactive appointment reminders increased the odds of visit attendance by 52% compared to passively receiving 2wT reminders. Higher fidelity of motivation message delivery had a small but independent effect on on-time clinic attendance. Receiving a missed appointment reminder did not significantly increase the odds of timely return to care. While participants generally received all core intervention components as intended, our in-depth analysis of implementation fidelity unveiled several notable challenges that warrant attention for optimizing implementation fidelity during 2wT expansion.

First, our evaluation of implementation fidelity highlighted that both instances of 2wT platform failure to transmit and to deliver messages occurred throughout the study. The former predominantly stemmed from 2wT technological issues, but was sustained by a lack of robust processes for monitoring platform performance. Notably, starting in the first week of April 2022, for five consecutive weeks, the 2wT platform failed to transmit motivation messages and appointment reminders as expected. This technical glitch, regrettably, remained undetected by the study team until participants alerted 2wT staff. This highlights the importance of continuous monitoring for rapid identification and prompt resolution of technological problems, as previously recommended.^[35]^ Following the detection of the technical glitch, a monitoring system was implemented in which the SMS aggregator shared the number of messages sent out by the platform with 2wT staff on a weekly basis. Reduced gaps in message transmission over time likely reflects improved monitoring practices introduced during the study. In the future, the enrolment of dummy phone numbers or the creation of process management dashboards visualizing performance metrics could help to alert implementers to platform underperformance and trigger swift intervention. Additionally, message delivery success varied across and within participants. Non-delivery likely resulted from individual user connectivity challenges rather than geographic connectivity differences.^[36–40]^ User behaviors, like SIM card swapping to compensate for telecom service coverage variations between service providers and varying network quality, phone switch off, phone loss, and dead batteries may also have contributed.^[38, 41, 42]^ The decline in the delivery of transmitted appointment reminder messages throughout follow-up may also reflect loss of participant interest or phone number changes over time,^[43]^ emphasizing the need for strategies to promote continued participant engagement. Despite these challenges, the majority of participants were reminded of each clinic appointment and missed clinic appointment indicating that the message delivery schedule (3 days and 1 day before scheduled appointments, and 2, 5, and 11 days following a missed appointment) was sufficient to offset connectivity challenges.

A second challenge identified through fidelity evaluation was the large proportion (> 50%) of missed appointment reminders that were sent mistakenly – wasting resources. Receiving unnecessary or incorrect reminders could potentially lead to participant annoyance, as has been reported previously.^[40]^ Unnecessary reminders likely resulted from the need to manually transfer appointment data between the EMRS and 2wT system, resulting in delays. In an attempt to mitigate this, the timing of the first reminder message was changed from one to two days after a missed appointment, providing more time to transfer the data manually. The increase in missed appointment message delivery seen throughout follow-up likely reflects optimization of these manual data transfer processes. Previous research has identified bi-directional data transfer between EMRSs and mHealth interventions as key to optimizing intervention impact and preventing data system fragmentation.^[44]^ Although integration of 2wT with the EMRS was not feasible during the study period, 2wT and EMRS data systems have since been integrated, reducing 2wT inaccuracies and enhancing efficiency. Integration should also mitigate participant frustration associated with receiving undeserved missed appointment reminders after reporting on time to clinic.

Finally, our implementation fidelity assessment uncovered that only a minority of affirmative responses to automated prompt inquiring about transfer or visit change requests resulted in documentation of care transfer or modifications to clinic appointments. This might be explained by participants having difficulty navigating multi-layer prompts. Automated prompts with ‘1’/’0’ response options are an efficient method of soliciting information and appeared to work well for participants confirming visit attendance. However, for participants responding, ‘0’ (no), the high proportion of false positives suggests that the response format may have been too rigid or that the prompt was not clear enough. Many 2wT participants instead responded with free text (outside the suggested ‘1’/’0’ response options) and engaged directly with 2wT personnel via SMS or requested phone calls to communicate their appointment change requests. This is in line with another mHealth study in Uganda, which identified poor participant understanding of the required response format as a barrier to achieving the high quality data needed for implementation decision making.^[45]^ In the study, up to 10% of participant responses could not be processed by automated systems as intended due to participants entering them in an unstructured, conversational format, resulting in a substantial unanticipated data-cleaning burden.^[46, 47]^ Simplification of prompt flows and strengthening of 2wT client education may improve 2wT data quality in the future.^[48]^ For those participants with smartphones, the potential of voice notes and emojis is also worthy of consideration.

Analysing implementation fidelity also facilitated hypothesis generation about which intervention components drive improved retention among 2wT participants. First, it appears that receiving an SMS reminder is an important trigger that makes participants aware of upcoming appointments and mobilizes them to go to the health facility, a finding in line with previous studies demonstrating the ability of text messages to increase appointment attendance.^[49–51]^ While not all two-way text message interventions are successful in improving attendance over standard of care or one-way text messaging, ^[52]^ in the field of medication adherence, the ability to respond to mHealth messages has been found to allow for tailored follow-up action and empower participants to take on a more active role in their own care.^[43, 53]^ Regarding the 2wT motivation messages, we had hypothesized, based on findings from our 2wT intervention human-centered design process, that weekly general health messaging could enhance care engagement by nurturing a sense of connection between participants and the clinic during inter-appointment intervals.^[32]^ In a study on mother-to-child HIV transmission, mothers living with HIV who received a text message containing general encouragement described how the message made them feel cared for, lifted their morale and fostered a sense of connection and acceptance that helped them engage in HIV care.^[54]^ Likewise, in a study among patients with type 2 diabetes, receiving SMS adherence messaging made participants feel like they had a supportive relationship with the sender, as if someone was looking out for them.^[55]^ Taken together, these findings suggest there may be a relational benefit of motivation messages that is independent from message content.

Our findings are not without limitations. Fidelity was conceptualized largely as message delivery success and evaluated mainly from the implementer’s, as opposed to the beneficiary’s perspective. The collected data did not allow for verification of whether messages were received by the intended study participants. Further study should explore whether messages reached the right person and the timing of that reach. Second, to assess fidelity of missed appointment reminders, visits reported in the EMRS were considered the gold standard. Although the Malawi EMRS has decades of evidence of quality data and Lighthouse Trust has a generator to prevent EMRS service breaks, it is possible that these errors in 2wT missed appointment reminders resulted from EMRS or other data weakness and not from 2wT system issues, suggesting the need for additional study of this fidelity measure. Third, we have attempted to characterize implementation fidelity through medians and totals. We recognize, however, that implementation is a dynamic process, and that implementation fidelity may vary over time and with different phases of the study. This dynamic nature is insufficiently captured in the current analysis. Additionally, gathering qualitative data would be a valuable addition to this evaluation of implementation fidelity. Interviews with study staff and study participants would aid in the assessment of implementation fidelity moderators such as intervention complexity and quality of delivery as well as shed a light on barriers and facilitators of implementing 2wT with fidelity, potentially identifying reasons for unsuccessful message delivery. Lastly, our evaluation of 2wT implementation fidelity was performed as a retrospective analysis within the research context. The complexity and intensive analysis of this investigation may not be replicable in routine, low-resource settings.

## CONCLUSIONS

Implementing evidence-based interventions with fidelity is central to achieving desired health outcomes. In order to optimize implementation fidelity, it first needs to be measured. Frameworks like the CFIF can structure these assessments and aid in the identification of facets of fidelity to consider. Our findings show that greater 2wT implementation fidelity (delivery of expected motivation messages and appointment reminders) was associated with improved clinic attendance. The majority of identified failures in the transmission of appointment reminders and missed appointment reminders was attributable to temporary poor platform performance, an implementation weakness exacerbated by lack of close system monitoring. Monitoring improvements implemented after this issue was detected reduced subsequent gaps in implementation fidelity. Monitoring of implementation fidelity should be integrated in study design so that systems can be put in place to swiftly alert implementers to deviations in intervention performance.

## Supplementary Material

Supplement 1

## Figures and Tables

**Table 1:** Fidelity by intervention core component

	EXPECTED	ACHIEVED
**Weekly motivation messages**	Participant receives a motivation message for 100% of weeks enrolled in the study	Participants received a motivation message for 75% (IQR 56% − 83%) of weeks enrolled in the study
**Proactive appointment reminders**	Participant receives a proactive reminder before 100% of appointments	Participants received a proactive reminder before 83% (IQR 67% − 100%) of appointments
**Missed appointment reminders**	Participant receives a reminder after 100% of missed appointments	Participants received a reminder after 67% (IQR 0% − 100%) of missed appointments
**Ability to report transfers and reschedule appointments**	Participant is able to report transfers through 2wT Participant is able to reschedule appointments through 2wT	Participants reported 47 transfers in response to automated 2wT prompts, of which 9 (19%) were true transfers upon follow-up Participants made 115 rescheduling requests in response to automated 2wT prompts, of which 46 (40%) resulted in appointment changes Participants rescheduled 196 appointments through unprompted 2wT interaction

**Table 2:** Mixed-effects logistic regression analysis of the effect of core component implementation on clinic attendance

		OR	95%L	95%H	P-value
**On-time clinic attendance**					
Univariable	10% increase in the percentage of enrolled weeks for which motivation messages were delivered	1.13	1.06	1.20	< 0.001
Univariable	Proactive appointment reminder receipt	1.83	1.44	2.33	< 0.001
Univariable	Appointment reminder response (any answer)*	1.52	1.19	1.95	< 0.001
Multivariable	10% increase in the percentage of enrolled weeks for which motivation messages were delivered	1.11	1.03	1.19	0.004
	Proactive appointment reminder receipt	1.65	1.29	2.12	< 0.001
Multivariable	10% increase in the percentage of enrolled weeks for which motivation messages were delivered	1.11	1.02	1.21	0.01
	Appointment reminder response (any answer)	1.5	1.17	1.93	0.001
**Return to care within 14 days of a missed appointment**					
Univariable	10% increase in the percentage of enrolled weeks for which motivation messages were delivered	1.12	1.03	1.22	0.01
Univariable	Missed appointment reminder receipt	1.33	0.91	1.95	0.14
Univariable	Missed appointment reminder response (any answer)*	1.48	0.86	2.55	0.15
Multivariable	10% increase in the percentage of enrolled weeks for which motivation messages were delivered	1.08	0.99	1.19	0.08
	Missed appointment reminder receipt	1.17	0.77	1.76	0.46
Multivariable	10% increase in the percentage of enrolled weeks for which motivation messages were delivered	1.03	0.90	1.17	0.72
	Missed appointment reminder response (any answer)	1.48	0.86	2.54	0.16

* Among those who received a reminder

## Data Availability

The data that support the findings of this study are included in supplemental materials (see Additional file 2).

## Abbreviations

2wT: Two-way texting
ART: Antiretroviral therapy
CFIF: Consolidated Framework for Implementation Fidelity
CHT: Community Health Toolkit
CI: Confidence interval
eHealth: Electronic health
EMRS: Electronic medical record system
IQR: Interquartile range
LMIC: Low- and middle-income countries
LT: Lighthouse Trust
mHealth: Mobile health
MPC: Martin Preuss Centre
OR: Odds ratio
PLHIV: People living with HIV
SMS: Short message service
SoC: Standard of care
