# Supplementary material for: Looking under the hood of a hybrid two-way texting intervention to improve early retention on antiretroviral therapy in Malawi: an implementation fidelity evaluation
Source: Res Sq. 2024 Dec 19:rs.3.rs-4965561. Preprint. [Version 1] doi: 10.21203/rs.3.rs-4965561/v1 (PMC11702799; doi:10.21203/rs.3.rs-4965561/v1)
Supplement: Supplement 1 [file NIHPPRS4965561v1-supplement-1.pdf]

Supplementary Files

This is a list of supplementary files associated with this preprint. Click to download.

- Additionalfile1.pdf
- Additionalfile2.xlsx
- Appendix1.csv
- Appendix2.pdf
